# Response of tibialis anterior tendon to a chronic exposure of stretch-shortening cycles: age effects

**DOI:** 10.1186/1475-925X-8-12

**Published:** 2009-06-29

**Authors:** James S Ensey, Melinda S Hollander, John Z Wu, Michael L Kashon, Brent B Baker, Robert G Cutlip

**Affiliations:** 1 National Institute for Occupational Safety and Health (NIOSH), Health Effects Laboratory Division, Morgantown, West Virginia 26505, USA

## Abstract

**Background:**

The purpose of the current study was to investigate the effects of aging on tendon response to repetitive exposures of stretch-shortening cycles (SSC's).

**Methods:**

The left hind limb from young (3 mo, N = 4) and old (30 mo, N = 9) male Fisher 344 × Brown Norway rats were exposed to 80 maximal SSCs (60 deg/s, 50 deg range of motion) 3x/week for 4.5 weeks *in vivo*. After the last exposure, tendons from the tibialis anterior muscle were isolated, stored at -80°C, and then tested using a micro-mechanical testing machine. Deformation of each tendon was evaluated using both relative grip-to-grip displacements and reference marks via a video system.

**Results:**

At failure, the young control tendons had higher strain magnitude than the young exposed (p < 0.01) and the old control tendons (p < .0001). Total load at inflection was affected by age only (p < 0.01). Old exposed and control tendons exhibited significantly higher loads at the inflection point than their young counterparts (p < 0.05 for both comparisons). At failure, the old exposed tendons carried higher loads than the young exposed tendons (p < 0.05). Stiffness was affected by age only at failure where the old tendons exhibited higher stiffness in both exposed and control tendons than their young counterparts (p < 0.05 and p < 0.01, respectively).

**Conclusion:**

The chronic protocol enhanced the elastic stiffness of young tendon and the loads in both the young and old tendons. The old exposed tendons were found to exhibit higher load capacity than their younger counterparts, which differed from our initial hypothesis.

## Introduction

Physical activity-induced tendinopathies are a common occurrence in athletes, with higher incidence in those more involved in sports and activities that require high physical demand [1]. It is also clear that aging exacerbates the susceptibility to injury during physical activities [1]. However, there is a paucity of information regarding injury mechanisms in tendon and appropriate countermeasures to increase tendon performance and decrease injury in an aging population. Tendons transmit forces from muscles to bones and experience much higher stress during locomotion than any other components in the musculoskeletal system [2]. Many researchers study the mechanical characteristics of mammalian tendons [3-8] and have yielded insight into the baseline elastic and viscoelastic properties in both animals and humans. Typical elastic tendon properties are characterized by Young's modulus and stiffness. Young's modulus is classically defined as the modulus of elasticity (E) of a material calculated by the rate of change of stress with strain and is an intrinsic property. Stiffness is the resistance of an elastic body to deformation by an applied force, typically defined by the ratio of change in tensile force with change in length of the material, thus an extrinsic property.

Repetitive mechanical loading can predispose tendons to injury with damage initiation occurring in the extracellular matrix [9-11]. The accumulation of micro-damage in tendons tends to degrade their mechanical properties, and may ultimately lead to failure. However, tendons can adapt to mechanical usage as evidenced by increases in stiffness and the Young's modulus after strength training or a combination of resistance and stretch training that were commensurate with muscle strength and size gains in humans [3,12] and in animals [10]. Also, tendon stiffness and ultimate strength have also increased in response to endurance training [6,13]. Viidik examined the response of rabbit tibialis anterior and Achilles tendon to 40 weeks of treadmill exposure and reported an increased stiffness of 10% in both tendons [13]. Nielsen et al. [14] also studied the effects of 18 months of treadmill training on rat limb muscle tendons and found that exercise had no effect on the biomechanical properties of the tibialis anterior tendon. Simonsen et al. [6] investigated whether tendon would respond differently to resistance or endurance training regimens in rats. Their results indicated that strength training did not result in increases in ultimate strength; however, swim-trained rats did have tendons with significantly higher ultimate strength than age-matched controls. The authors suggested that tendon may respond more favorably to the number of cycles of loading rather than the magnitude of loading [6]. This was supported by findings from Buchanan and Marsh where treadmill exposure for 8–12 weeks was found to increase tendon stiffness in the Achilles tendon of guinea fowl [10]. This result was reinforced in humans where long distance runners exhibited significant increases of approximately 20% in vastus lateralis stiffness compared to control subjects [15]. However, exposure to stretch training alone did not increase stiffness in human tendons [16].

As we age, it is not surprising that tendon properties such as stiffness and Young's modulus can change along with other physiological changes [7,17,18]. There is an increase in tendon strength up to a certain age, where tendon properties then start to degrade [19]. In fact, investigators found that the strength of 23 month-old rat tail tendons was higher than those from 5 month-old rats [19]. In another study, Nielsen and colleagues found that aging rendered the rat tibialis anterior tendons stiffer and reduced the strain to failure [14]. In contrast to the findings by Viidik et al. and Nielsen et al., Simonsen and colleagues found that aging reduced the ultimate failure force and yield point in rat Achilles tendon [6]. However, tendons in aging subjects have been shown to be highly responsive to training. Specifically, resistance training increases stiffness and Young's modulus [7,8,17,18,20], and decreases hysteresis [18] in older humans. These results in humans were supported by studies conducted in rats [21]. Also, Simonsen found that swim training counteracted the negative influence of aging on Achilles tendon strength [6]. In contrast, chronic running exercise did not benefit the musculo-tendon unit in aged runners [22].

Stretch-shortening cycle (SSC) exercise effectively introduces resistance exercise to skeletal muscle [23] via reciprocal concentric and eccentric muscle actions which are physiologically representative of natural muscle function used in common activities such as locomotion, and in athletic and occupational environments [24,25]. Additionally, SSCs also produce muscle injury due to the eccentric component of the loading cycles [26-30], which provides an improved physiologically relevant exposure model over the traditional eccentric-only injury model [24,31]. Recently, a chronic exposure (14 exposures) of repetitive SSCs was shown to produce skeletal muscle hypertrophy and significant muscle performance gains in young rats (12 weeks age) while inducing substantial performance deficits and a lack of muscle hypertrophy in old rats (30 months age) after 4.5 weeks of exposure [23]. This study showed that muscles from aging rats did not tolerate exposure to repetitive mechanical loading that is beneficial in their younger counterparts. Thus, it would be interesting to investigate whether tendon from aging rats also does not tolerate this repetitive loading protocol that resulted in muscle maladaptation.

To date, there is little known about the effects of resistance exercise and ageing on tendon mechanical properties. The resistance training paradigms studied in humans [7] and animals [21] thus far have resulted in improvements in both muscle and tendon; however, the biomechanical loading was not controlled or recorded during the exposures. In addition, the results from previous studies are not equivocal. Thus, it is important to study tendon response to a chronic exposure of repetitive maximal SSCs, shown to produce muscle maladaptation in aged animals, where the biomechanical loading signature is controlled, and muscle response is recorded in real-time. The purpose of this study is to determine if aging affects the ability of tendon to respond to repetitive high-force mechanical exposures. This inquiry will help determine if tendon adaptation is coupled with skeletal muscle response. We hypothesize that tendons from old rats not exposed to repetitive loading will have lower stiffness, Young's modulus, and total strain at failure than their younger counterparts. In addition, we hypothesize that exposure to repetitive mechanical loading will increase the stiffness, Young's modulus, and strain at failure in both old and young tendons.

## Methods

### Animal exposure and sample preparation

We obtained male Fischer Brown Norway Hybrid Rats (F344 × BN F1) from the National Institute of Aging colony. Young adult (N = 4, 305 ± 26 g standard deviation (SD), 3 months of age) and old (N = 9, 530 ± 38 g SD, 30 months of age) rats were housed in an Association for Assessment and Accreditation of Laboratory Animal Care (AAALAC) accredited animal facility where temperature, humidity, and light/dark cycles were held constant for all rats; food and water were provided ad libitum. We allowed the rats to acclimate for one week before beginning the chronic exercise exposures that were approved by the NIOSH Animal Care and Use Committee [23].

In this study, we exposed the left dorsiflexor muscles of young and old rats to 8 sets of 10 repetitions of maximal force stretch-shortening contractions (SSCs) with 2-min intervals between sets using a custom-designed dynamometer, while the right contralateral limb served as control [32]. Within each set, there was a 2-s rest between SSCs. For each repetition, an electrical stimulator fully activated the dorsiflexor muscles for 100 ms duration. The eccentric contraction phase was initiated with a 60 deg/s movement velocity of the load cell fixture over the prescribed range of motion of 90–140 deg ankle angle. The load cell fixture was then immediately returned in the concentric phase, at 60 deg/s, to the starting position of 90 degs ankle angle. The dorsiflexor muscles were deactivated 300 ms later. Total stimulation time per repetition was 2.06 s. We administered the SSC protocol three times per week over a 4.5 week period for a total of 14 exposures (Appendix 1, step 1). We designed the exposure paradigm based on findings from a previous study that indicated that this protocol produced significant hypertrophy and performance gains in the young rats and performance loss and absence of hypertrophy in the old rats [23].

Twenty-Four hours after final exposure, we weighed, anesthetized with sodium pentobarbital (ip, 10 mg/100 g BW) and euthanized the rats by exsanguination. We isolated the tibialis anterior tendons of the right and left hind limbs and placed them in phosphate-buffered saline (PBS) for storage at -80°C (Appendix 1, step 2). Previous studies have found that this freezing preservation has negligible effects on the elastic and viscoelastic properties of the tendons [33].

### Experimental set-up

We clamped each tendon specimen between two custom-metal grips which are composed of fixed blocks and sliding grips. The grips slid in the v-shaped slots of the fixed blocks during pulling, such that the gripping force increases with increasing stretch force. In order to increase the friction force between the tendon and grips, we covered the contact surfaces with fine sand paper (grit # 200). The tests were performed using a universal micro-mechanical testing machine (type Mach-I, Biosyntech, Montreal, Canada). The testing machine was equipped with a displacement sensor with a resolution of 0.5 microns and a 98 N (10 kg) load cell with a resolution of 4.50 mN (500 mg). Reference marks were made on the tendons using permanent ink before they were installed in the test fixture (Appendix 1, step 3). We submerged each tendon specimen and the grips in PBS solution at room temperature (22°C)(Appendix 1, step 4). In order to eliminate the errors due to the relative sliding between the grips and the tendon specimen, we measured the tendon deformation visually via a video system. The tendon deformation is quantified by determining the position variations of the reference markers on the specimen. Since the resolution of the position recognition of the tendon mark was limited, we used two marks with the maximal distance between them, i.e., the marks near the clamping sites, as the reference marks to minimize measurement error [34].

The distance between the grips was approximately 7 mm, and the distance between the reference marks used for the data processing was approximately 5 mm. We evaluated the deformation of the specimens using the relative displacements between the grips, and also between the reference marks.

The microscope video system included a color CCD camera (JAI, Woburn, Massachusetts, USA) and a microscope video lens (Infinity Photo-Optical Company, Boulder, Colorado, USA). LabView IMAQ Vision software, a PCI-1422 Framegrabber, and an AI-16XE-50 DAQCard (National Instruments, Houston, TX, USA) were used to record the displacement of the tendon marks during the loading. A customized user interface provided calibration, resolution validation, image capture, and image post-processing routines. One of the DAQ Card counters triggered the buffered image acquisition at a rate of 10 Hz. Prior to the tendon experiments, we tested the timing and system capabilities using a rubber band. Displacement time-histories of the reference marks were obtained using pattern recognition algorithms with region-of-interest subroutines to eliminate error due to false positive recognitions.

### Tendon test procedure

In order to obtain repeatable test results, each tendon specimen was preconditioned via sinusoidal, cyclic loading of six cycles for 60 seconds, which was performed using a displacement protocol with a peak strain magnitude of approximately 0.2% (Appendix 1, step 5). Each tendon was then relaxed for 30 seconds (Appendix 1, step 6), and stretched up to failure at a loading speed of 1 mm/s (Appendix 1, step 7). The tendon strain was defined as the tendon deformation (i.e., the relative displacement between the reference marks) divided by the reference length (i.e., the distance between the tendon reference marks in the undeformed state). The reference length is defined as the measured length between the tendon markers when the force (above 1 gram) begins to increase with increasing displacement. The load data were recorded directly from the load cell on the testing apparatus during testing, and they were used in calculating the stiffness at the inflection and failure points of each sample.

**Figure 1:**
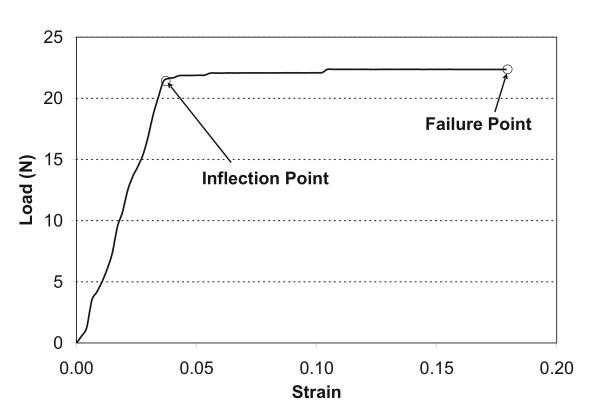
**An example of a load/strain curve with inflection and failure points indicated for one sample from an old animal**.

Fig. 1 shows typical load-strain curves of the tendon. Load, measured in Newtons (N), increases as the strain increases, until it reaches a plateau and subsequent failure. We calculated strain by using the tendon marks from camera images in the undeformed state and at the last image of the tendon at the final moment before failure for each sample. We defined the point where the load ceases to increase and plateaus as the inflection point which was recorded along with the load and strain corresponding to the failure point. These plots were then used to determine the inflection point, where the tendon moves from elastic deformation to plastic deformation. In the test, we determined the inflection point graphically as the cross point of the two tangential lines around the inflection point of the curve, as shown in Fig. 1. The stiffness was then determined for this point, as well as for the failure point by dividing the change in load by the change in strain. The means and standard error of mechanical strain, load, and stiffness were also calculated for each group.

### Statistical analysis

Tendon data were analyzed using SAS/STAT software, Version 9.1 of the SAS System for Windows (SAS Institute, Cary, NC). A mixed model two-factor within-subject analysis of variance (ANOVA) was used to conduct the initial statistical analysis. The design factors included age and loading treatment (i.e. stretch-shortening cycle treatment or limb). Since tendons from both limbs were assessed, animal was included as a random effect to appropriately model the covariance structure. Data that were normalized to the untreated limb were analyzed using one-way ANOVAs with age as the factor. Post hoc comparisons were also carried out using Fishers Least Significant Difference method. All differences and effects were considered significant if p < 0.05. All data are depicted as the mean value ± standard error.

## Results

**Figure 2:**
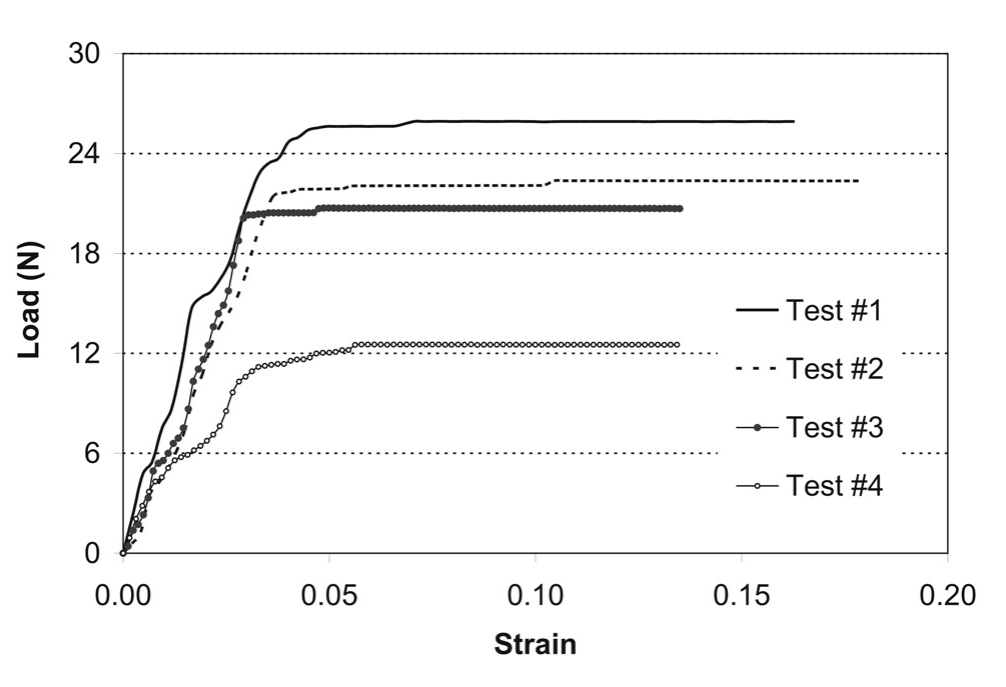
**Typical load/strain curves from the loaded limb of one group of four old rats**.

The load/strain curve of young tibialis anterior (TA) tendons followed a typical pattern for elastic materials (Figure 1). The inflection and failure points are noted in the typical response pattern. Tendons from the exposed limbs of the old rats followed similar patterns as the young tendons when loaded to failure (Figure 2).

### Strain

**Figure 3:**
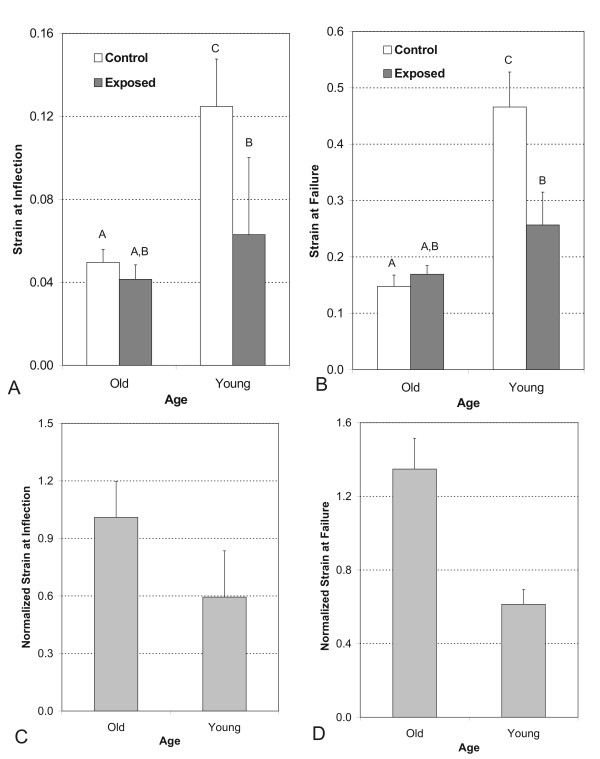
**(A) Mechanical strain of tendons from loaded versus unloaded limbs by age group at the inflection point**. (B) Mechanical strain of tendons from loaded versus unloaded limbs by age group at the failure point. (C) Normalized mechanical strain for old and young tendons at the inflection point. Each loaded tendon is normalized to its unloaded control. (D) Normalized mechanical strain for old and young tendons at the failure point. Each loaded tendon is normalized to its unloaded control. Data is depicted as mean values ± standard error. Different letters denote significance at the 0.05 level.

The total strain magnitude at the inflection point of the TA tendons was affected by age (p < 0.01) and exposure (p < 0.05, Figure 3A). There was no difference in strain magnitude at the inflection point between the control (0.049 ± 0.012) and exposed tendons in the old rats (0.041 ± 0.011). In the young rats, tendons from the control limb (0.1248 ± 0.020) exhibited a significantly larger strain magnitude than tendons from the exposed limb (0.062 ± 0.017, p < 0.05). The strain magnitude at the inflection point in tendons from the exposed limb of the old rats differed little from that of the young rats, while tendons from the control limb of the young rats exhibited a larger strain magnitude than those of the old rats (p < 0.01, Figure 3a).

The strain magnitude to failure of the TA tendons was affected by age (p < 0.0001, Fig 3B). Also, the tendons from the old rats responded differently with exposure to loading than the young rats as evidenced by an age *x *limb interaction (p = 0.0020). In old rats, the failure strains in tendons from the exposed limbs (0.1692 ± 0.023) were not different than those from the control limbs (0.1477 ± 0.025); while in the young rats, tendons from the control limb (0.4660 ± 0.041) had higher failure strains than the tendons from the exposed limb (0.2567 ± 0.035, p < 0.01). Furthermore, the tendons from control limbs of young rats had significantly higher failure strains than those from control limbs of old rats (p < 0.0001). Tendons from exposed limbs of young and old rats did not exhibit an age effect although it was approaching significance (p = 0.0564, Figure 3B).

The normalized strain (strain of exposed limb tendon/strain of control limb tendon) at the inflection point for the old and young rats was 1.00 ± 0.86 and 0.59 ± 0.24, respectively (Figure 3C). At the failure point, the normalized strain was 1.34 ± 0.16 and 0.61 ± 0.08 for the old and young tendons, respectively (Figure 3D). Thus, at failure the old rats exhibited larger strains to failure after exposure to the chronic resistive exercise. In contrast, the young rats responded to the exercise protocol by exhibiting less strain to failure.

### Load

**Figure 4:**
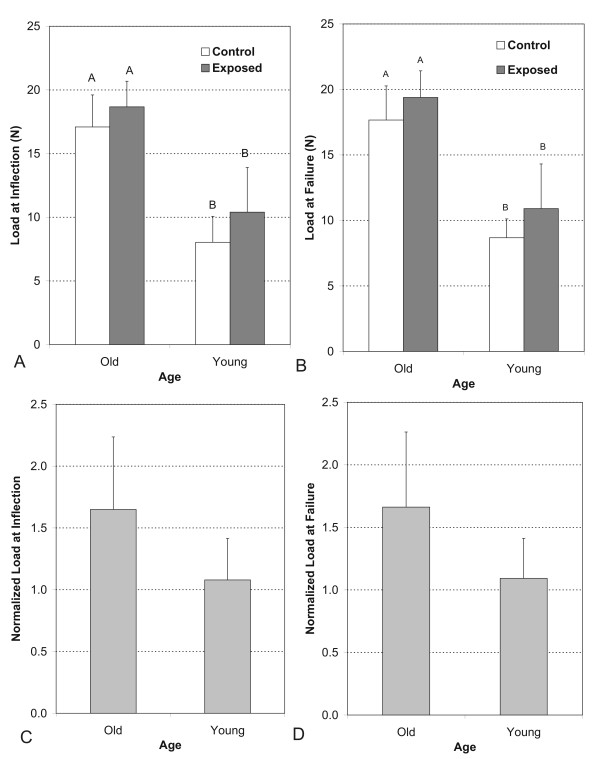
**(A) Mechanical load of tendons from unloaded and loaded limbs at the inflection point by age group**. (B) Mechanical load of tendons from unloaded and loaded limbs at the failure point by age group. (C) Normalized mechanical load for old and young tendons at the inflection point. Each loaded tendon is normalized to its unloaded control. (D) Normalized mechanical load for old and young tendons at the failure point. Each loaded tendon is normalized to its unloaded control. Data is depicted as mean values ± standard error. Different letters denote significance at the 0.05 level.

The total load at the inflection point was affected by age (p < 0.001) but the exposure protocol had no effect on the response of the tendons. In the exposed limb, tendons from old rats (18.67 ± 2.11 N) exhibited higher loads at the inflection point than tendons from young rats (10.40 ± 3.17 N, p = 0.0426). In the control limb, tendons from old rats (17.09 ± 2.24 N) also exhibited significantly higher loads at the inflection point than tendons from young rats (8.03 ± 3.66 N, p < 0.05) (Figure 4A).

The total load at failure was affected by age (p < 0.001), but not by the chronic exposure protocol. In the exposed limb, tendons from old rats (19.38 ± 2.14 N) had higher failure loads than those from young rats (10.90 ± 3.21 N, p < 0.05). In the control limb, tendons from old rats (17.66 ± 2.27 N) had higher failure loads than those from the young rats (8.71 ± 3.71 N), (p = 0.05, Figure 4B).

The normalized load (load of loaded limb tendon/load of unloaded limb tendon) at the inflection point was 1.64 ± 0.58 and 1.07 ± 0.33 for the old and young tendons, respectively (Figure 4C). The normalized load at the failure point was 1.66 ± 0.06 and 1.09 ± 0.31, respectively, for the old and young tendons (Figure 4D). At the failure point, the results were similar to the inflection point, where the old rats responded positively to the chronic exercise protocol by increasing their load to inflection and failure as compared to their non-exposed tendons, while the young rats did not respond.

### Stiffness

**Figure 5:**
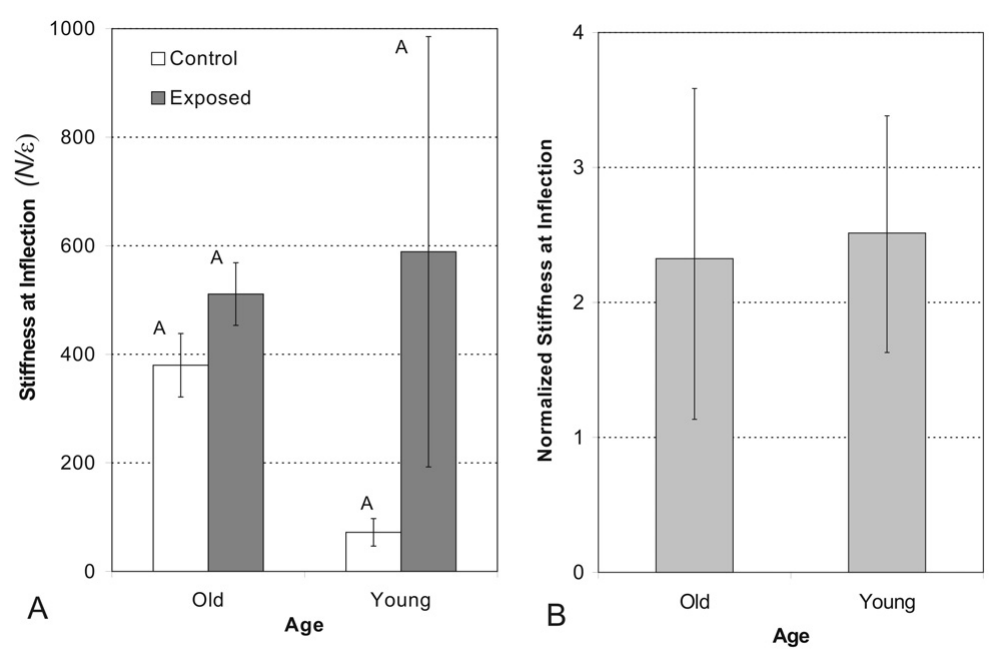
(A) The stiffness from unloaded and loaded limbs at the inflection point by age group. (B) The stiffness from unloaded and loaded limbs at the failure point by age group. (C) Normalized stiffness for old and young tendons at the inflection point. Each loaded tendon is normalized to its unloaded control. (D) Normalized stiffness for old and young tendons at the failure point. Each loaded tendon is normalized to its unloaded control. Data is depicted as mean values ± standard error. Different letters denote significance at the 0.05 level.

There was no significant difference between two age groups or limbs for stiffness at the inflection point (Fig 5A). The normalized stiffness (stiffness of exposed limb tendon/stiffness of control limb tendon) at the inflection point was 2.38 ± 1.26 and 2.44 ± 0.86 for old and young tendons, respectively (Fig 5B).

## Discussion

Adaptation of tendon in response to strength training has received little attention to date. Tendon is a viscoelastic material that has been shown to respond to mechanical loading derived from either strength or endurance training. Some studies have reported that tendon stiffness either decreases with aging [6,35-37], or does not change at all [38,39]. The mechanical stiffness and Young's modulus has been shown to decrease with increasing age in humans [8,35,40]. The lower Young's modulus indicates that it is due to intrinsically weaker tendon structures. A more compliant tendon in older adults can result in slower transmission of force, slower torque development, and decreased performance [7]. Usually, there is an increase in tendon mechanical properties until a certain age, after which the mechanical properties decrease [41]. Nielsen et al. also concluded that aging in rats renders tendons stiffer, increased the energy absorption, and also decreased their strain values at failure [14]. Our results support these findings and indicate a positive change with age up to 30 months in the Fischer Brown Norway hybrids. In this study, the young unexposed tendons had decreased stiffness as evidenced by more strain to both the inflection point and the failure point than the old unexposed tendons. The older unloaded tendons also withstood higher loads at both the inflection and failure points than the young unloaded tendons. This is also in agreement with prior results suggesting that tendons become stiffer with age [42]. Indeed, Achilles tendon from immature rabbits had less tensile strength than those from young adult and old rabbits due to the immaturity of the tendon in the young rabbits [40]. When physiological maturation is controlled for, clearly the trend is that mature tendons are stiffer and stronger than those from immature animals [40]. Our study examined 3 month old rats that may have immature connective tissue development. Also, the 30 month old rats of this species (Fischer Brown Norway hybrids) may have connective tissue development that is not in decline at this stage of the life cycle. Re-examining this study using a larger sample size would be beneficial to validating these results.

Resistance training has been shown to produce benefits in tendon mechanical properties much as it does to skeletal muscle shown in previous studies in animals [21,43-49] and humans [50]. Resistance training has also been shown to attenuate the negative effects of aging on muscle and tendon properties in humans by increasing their tensile stiffness and modulus [18,51,52]. The increased stiffness can impact the muscle's operating range as a stiffer tendon will allow less muscle shortening, causing a shift to a more optimal sarcomere overlap [7]. This can result in an increased rate of force and torque development. More recently, animal models have been developed to study the effects of resistance exercise and aging [6,53]. These models have also shown benefits of resistance exercise in reducing the effects of sarcopenia, glucose balance, bone loss, and other associated effects of aging. Increased tendon stiffness has been reported in animal studies where mechanical loading has been used to increase tendon load above normal physiological conditions [54,55]. In our chronic SSC exercise model, we recently showed that repeated exposures to SSCs resulted in muscle adaptation in young rats and maladaptation in old rats [23]. Using this same loading model, the exposed tendons from the young rats had lower strains to the inflection and failure points than the unloaded control tendons. Thus, this resistance protocol increased stiffness and reduced strain to failure in the young tendons, a positive effect. However, the tendons from the old rats did not change their elasticity and were not different from the unloaded control tendons. The loads at both the inflection and failure points were trending higher in the exposed versus the control tendons in both the young and old groups. Thus, the loading protocol may be producing some positive effects although the exposed tendons from the old rats exhibited higher load capacity than their younger counterparts. These results are not equivocal. Simonsen et al. did not find any positive benefits in Achilles tendon after 4 weeks of resistance training in aged animals, but did find a positive result with swim training [6]. Nielsen did not find a positive benefit from 18 months of running exercise in Sprague Dawley rats, but observed a positive benefit of aging and tendon mechanical properties [14]. Thus, all modes of exercise do not result in positive tendon adaptations.

It would be beneficial to explore whether mechanical properties of tendon change as rats mature to understand how aging affects tendon properties. The exposure protocol used did produce profound differences in skeletal muscle as previously reported [23]. The chronic protocol resulted in concurrent maladaptation of skeletal muscle and without maladaptation in tendon in the old rats which is a novel finding. Our thought is that as mechanical performance of the TA muscle declined during the chronic protocol, the forces did not overload the tendon, thus allowing for tendon adaptation in the old rats. Prolonged cyclical loading can produce deleterious effects in tendon than can be exacerbated by age [56], thus it is important to design the loading protocol not to exceed the tolerance limit of the tendon. While our previous work showed maladaptation of skeletal muscle in old rats after a chronic exposure of SSCs, the tendon response was shown to be quite different. This suggests that tendon and muscle can respond differently to a mechanical stimulus, and results from *in vivo *performance testing of the musculo-tendon system should not be used as the sole determinant of the adaptive or maladaptive kinetics of the tendon.

## Conclusion

These results may have particular application to training in elderly populations and implications to *in vivo *function. Maintenance of tendon mechanical properties in aging is vitally important for adequate performance of daily physical tasks where the utilization of muscular force and power is needed. Indeed, resistance training has been shown to produce positive benefits in tendon mechanical properties in humans and rodents, thus it is possible to maintain tendon performance into senescence with the appropriate use of exercise countermeasures.

## Competing interests

The authors declare that they have no competing interests.

## Authors' contributions

JSE conducted the data analysis and writing of the manuscript, MSH performed testing of the tendons, JZW designed and fabricated the tendon testing apparatus and assisted with data analysis, MLK performed the statistical analysis, BBB assisted in generating the introduction section, and RGC designed the experiments and assisted in writing the manuscript.

## Appendix 1 -

Tendon Exposure and Testing Protocol

1. Rats were exposed to a chronic loading protocol for 4.5 weeks.

2. Rats were euthanized and tendons were removed and stored at -80°C.

3. Tendons were thawed on morning of testing and loaded into clamps and marked with reference marks.

4. Clamped tendons were submerged in 1× PBS at room temperature and attached to a universal micro-mechanical testing machine.

5. Tendons were preconditioned by sinusoidal, cyclic loading of 10 cycles for 60 seconds with a peak strain magnitude of 0.2%.

6. Tendons were relaxed for 30 seconds.

7. Tendons were stretched to failure at a speed of 1 mm/s.
